# Supplementary figures and images for: Exploring equity in audit and feedback trials: Secondary analysis of a systematic review
Source: PLoS One. 2026 Mar 9;21(3):e0339361. doi: 10.1371/journal.pone.0339361 (PMC12970933; doi:10.1371/journal.pone.0339361)

### S1 Table: PRISMA-Equity Checklist


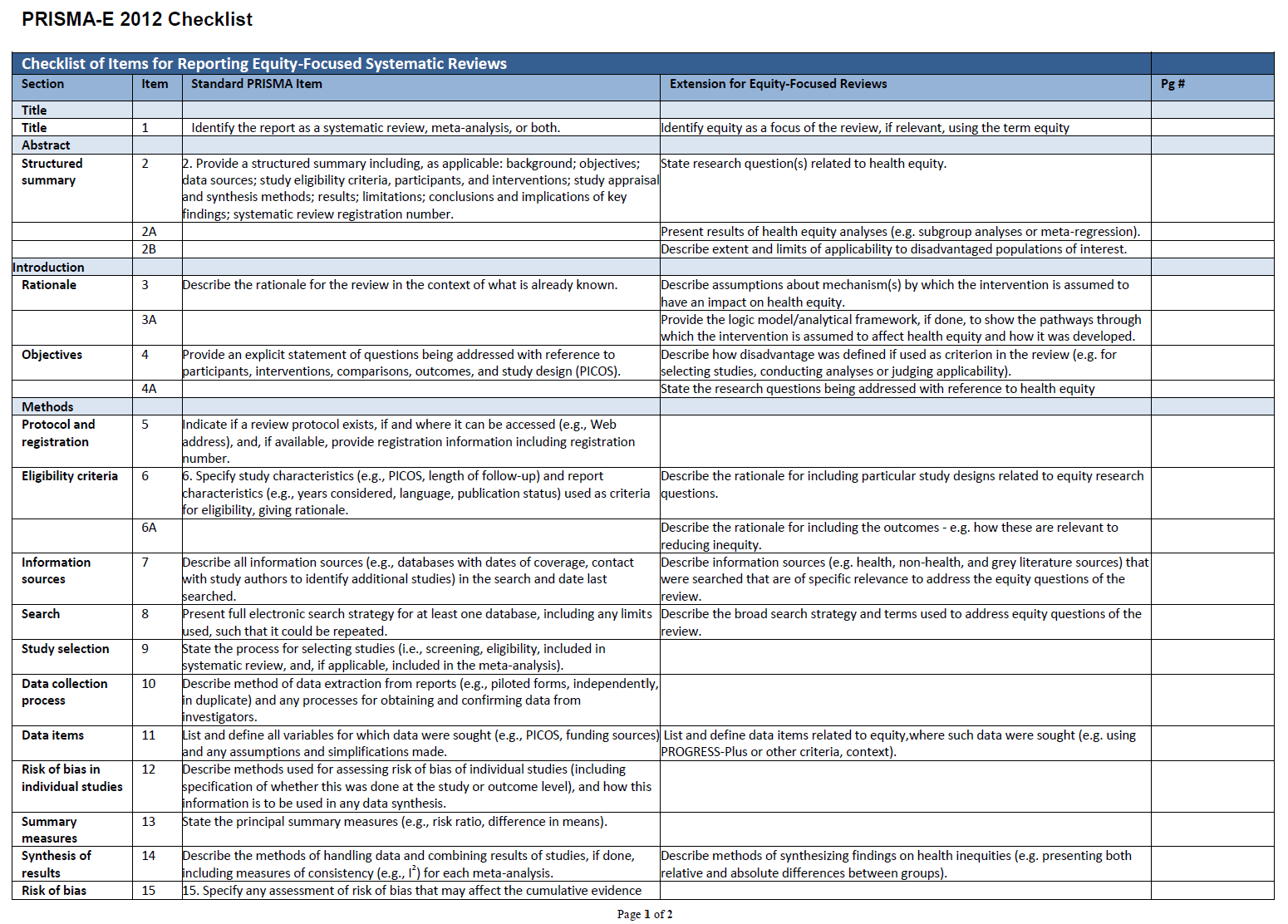


1

2-3

2-3

2-3

4

7-8

5-6

5-6

N/A

7

N/A

6, N/A

6, N/A

6

7-8

Appendix 2

N/A

N/A

7-8

N/A


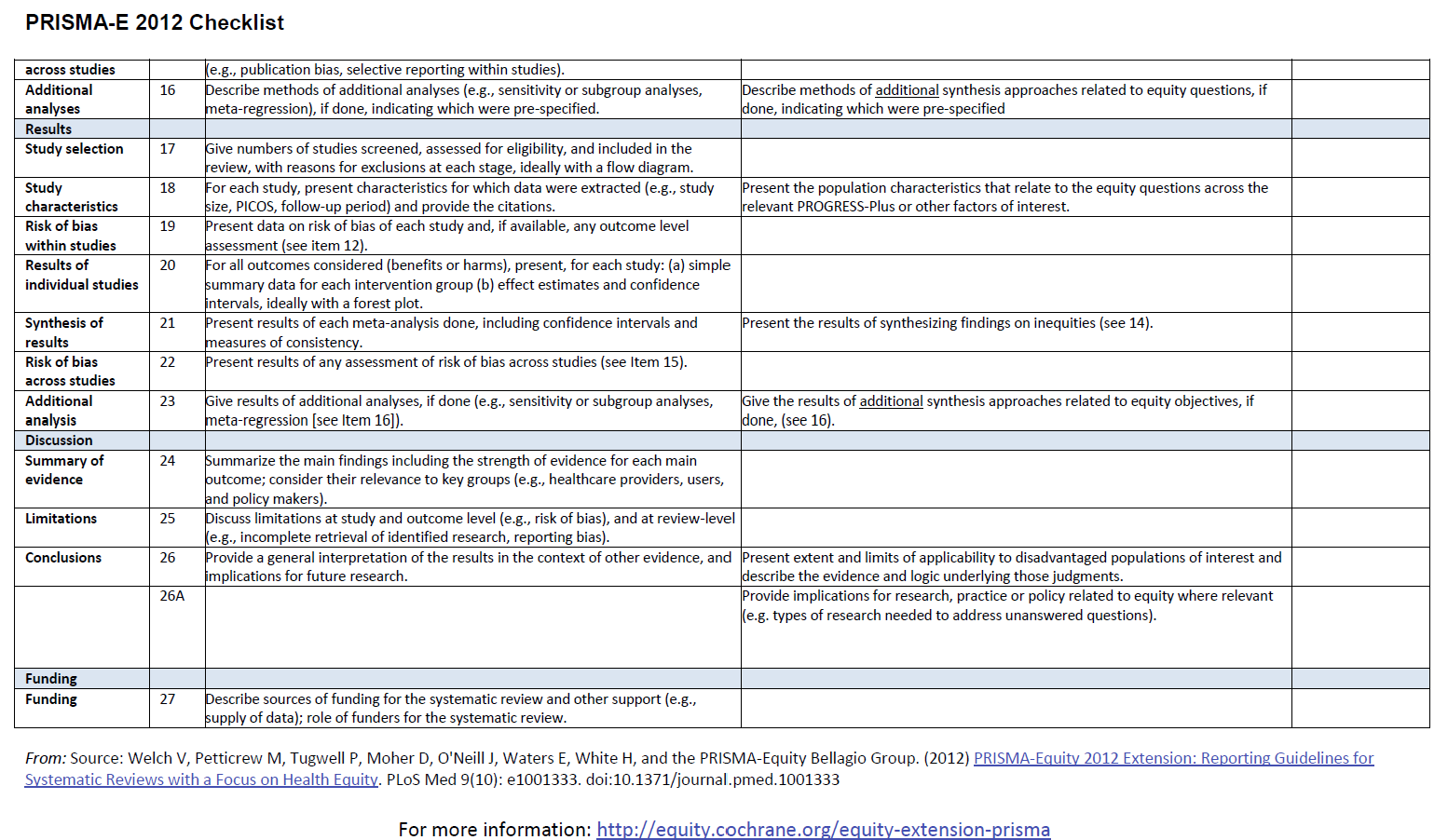


8

9-10

13-14

N/A

N/A

N/A

N/A

14-15

16-17

17-18

16-18

16-18

18

Supplement: S1 Table — (DOCX) [file pone.0339361.s001.docx]
