# Supplementary material for: Exploring equity in audit and feedback trials: Secondary analysis of a systematic review
Source: PLoS One. 2026 Mar 9;21(3):e0339361. doi: 10.1371/journal.pone.0339361 (PMC12970933; doi:10.1371/journal.pone.0339361)
Supplement: S2 Table — Adapted from Lu et al. (2018) Supplemental info. (DOCX) [file pone.0339361.s002.docx]

### S2 Table: Definition of PROGRESS-Plus Factors

*Adapted from Lu et al 2018 Supplemental info.*

| **PROGRESS-Plus Factor** | **Description and Notes** |
| --- | --- |
| Place of residence | Residence (for patients) or delivery of services (for providers) in an area considered ‘underserved’ or where it is difficult to access care. |
| Race/Ethnicity/Culture/ Language | Patients who do not identify with the dominant culture of the area due to not fluently speaking the dominant language of the country/region and/or being an ethnic, racial or cultural minority |
| Occupation | Occupational categories or classification that may affect the receipt or provision of health services |
| Gender/Sex | Gender and/or sexual roles or identities that may indicate differential access to health services, differential exposure to health risks or increased likelihood of violence and discrimination |
| Religion | Religious affiliations or beliefs that may lead to discrimination and bias from service providers or limit acceptability of or ability to choose certain health services |
| Education | Education level or education opportunities correlate with income status, knowledge about health and access to preventive health services/activities. |
| Socioeconomic Status | More direct measures of income levels. Income levels limit or facilitate access to health services (including preventive services), ability to pay for healthcare service |
| Social Capital | Social relationships and availability of social support networks to provide support and build resilience in time of illness or other distress |
| Plus | Factors that may limit a person’s ability to manage their health or seek/obtain healthcare; or factors that may lead to discrimination or bias from healthcare providers.   1. Personal characteristics associated with discrimination or stigmatizing experiences, e.g., age (old or young), in institutionalized care setting, disability, sexual orientation, proxy measures of SES, substance use (e.g., smoking) 2. Features of relationships, e.g., smoking parents, excluded from school, dependency on a caregiver 3. Time-dependent relationships, e.g., leaving the hospital, respite care, other instances where a person may be temporarily at a disadvantage. |

SES: Socioeconomic status
