# Supplementary material for: Exploring equity in audit and feedback trials: Secondary analysis of a systematic review
Source: PLoS One. 2026 Mar 9;21(3):e0339361. doi: 10.1371/journal.pone.0339361 (PMC12970933; doi:10.1371/journal.pone.0339361)
Supplement: S1 File — (DOCX) [file pone.0339361.s003.docx]

### S1 File: Data Extraction Form

1. **Key study characteristics [from main review]**: study ID, country of conduct, study setting(s), provider type, patient population/health issue, description of primary outcome (unit of analysis, clinical behaviour category, specific behaviour/outcome)
2. **Title**: equity-oriented verbiage, e.g., (in)equality, disparity, (in)equity, (un)fair or PROGRESS-Plus factors in the title
3. **Abstract**: intervention outcomes or participant demographics reported across (sub)groups defined by PROGRESS-Plus factors
4. **Background**:
   1. Framework, theory or conceptual approach used to guide consideration of equity in development and/or evaluation of the intervention
   2. Anticipated differences in baseline risk or intervention acceptability, coverage, or effectiveness across (sub)groups defined by PROGRESS-Plus
5. **Objective**: explicit equity-oriented study objective(s) and associated PROGRESS-Plus factors
6. **Inclusion/exclusion criteria**: participant (provider and/or patient) eligibility categories defined by PROGRESS-Plus factors
7. **Participant characteristics**: participant characteristics defined by PROGRESS-Plus factors
8. **Results/Analysis**: reported outcomes stratified by PROGRESS-Plus factors or reported outcomes where PROGRESS-Plus factors were used in the analysis
9. **Classification of study’s equity-orientation**:
   1. Not equity-oriented: study does not have an explicit equity-oriented objective AND no PROGRESS-Plus factors are used in the data analysis/results as stratifying variables
   2. Equity-informed: PROGRESS-Plus factors are used in the data analysis/results as stratifying variables BUT the primary outcome is not reported for a PROGRESS-Plus defined group
   3. Equity-focused: The study objective explicitly pertains to equity AND the entire study population is defined by a PROGRESS-Plus factor or a PROGRESS-Plus factor is used to stratify reporting of the primary outcome
10. **Intervention [from main review]**: brief description of the intervention including role of A&F, nature of the comparator/target, actor targeted for behavioural changes (patient, provider, team/organization)
11. **Recruitment and Participant Flow**: Differential recruitment (process or outcomes), attrition and/or concerns about adherence across PROGRESS-Plus factors
12. **Discussion:** applicability, generalizability, or external validity across PROGRESS-Plus factors

### 
