# Supplementary material for: Exploring equity in audit and feedback trials: Secondary analysis of a systematic review
Source: PLoS One. 2026 Mar 9;21(3):e0339361. doi: 10.1371/journal.pone.0339361 (PMC12970933; doi:10.1371/journal.pone.0339361)
Supplement: Attachment — Submitted filename: LadakZ_PONE-D-25-32703_ReviewerResponses_Nov12,2025.docx [file pone.0339361.s005.docx]

November 12, 2025

Dear PLOS ONE Editors,

Thank you for taking the time to consider and review our manuscript. Please find below a point-by-point response for each editorial and reviewer comment for manuscript #PONE-D-25-32703, “Exploring equity in audit and feedback trials: Secondary analysis of a systematic review” by Ladak et al. Page and line numbers refer to the tracked document.

| **Reviewer Comments** | **Response** | **Page No.** |
| --- | --- | --- |
| **Editorial** | | |
| 1. Please ensure that your manuscript meets PLOS ONE's style requirements, including those for file naming. | Formatting and style requirements have been updated | N/A |
| 2. Please include captions for your Supporting Information files at the end of your manuscript, and update any in-text citations to match accordingly. Please see our Supporting Information guidelines for more information: http://journals.plos.org/plosone/s/supporting-information. | Added at the end of manuscript | 23 |
| 3. If the reviewer comments include a recommendation to cite specific previously published works, please review and evaluate these publications to determine whether they are relevant and should be cited. There is no requirement to cite these works unless the editor has indicated otherwise. | N/A | N/A |
| 4. Please review your reference list to ensure that it is complete and correct. | Reviewed and updated to IEEE (square brackets) style | 19-22 |
| **Reviewer #1** | | |
| In the methods section under the study selection criteria, all dates put together are confusing, and I cannot be sure when the review started and ended. Also, the period when the articles were retrieved or the period when the articles included were considered is not clear. | Thank you for noting this confusion. We have removed this wording to avoid confusion. Readers can look to the updated systematic review for more details. | 6-7 |
| Again, under the results, you reported 276 articles included in the main review, but you did not give the total number of articles that were retrieved. | We have made this writing more clear to identify how many articles were retrieved in the main systematic review. | 10 |
| **Reviewer #2** | | |
| The main finding of the study is the description of audit and feedback (A&F) trials that include components of equity in the feedback content. It would have been helpful if the study had provided comparative information on how these trials differ from A&F trials without equity components. | Thank you for your comment. We believe we have highlighted these differences in the manuscript. Specifically, Table 2 describes how the equity-informed and equity-focused trial reporting differs from articles that were not equity-oriented. Table 3 also describes a sub-analysis of equity-focused trials highlighting how these trials explicitly report and embed equity in their interventions. As for data specific to trials within the updated systematic review, and their effectiveness based on included PROGRESS-Plus factors, we do not have this data readily available, but we hope this work encourages others to do additional analyses to further outline differences between trials that are equity-oriented and those that are not, and have emphasized this in our discussion. | 19 |
| It would also be helpful to offer a practical definition of what constitutes an A&F initiative with equity components. For example, age distribution may reflect a potential equity issue, or it may simply represent the inherent characteristics of the underlying disease studied or the eligible patient population. | Thank you for this comment. We acknowledge the subjectivity of what constitutes an A&F initiative that is equity-oriented and have explained this challenge in our discussion (lines 284-298). We believe our definitions of not equity-oriented, equity-informed, and equity-focused to explain what we defined in our analysis as constituting an equity-oriented A&F initiative. We have provided further explanation in our methods (lines 183-188). | 9 |
| The study does not provide information whether the equity-focused or informed A&F interventions lead to changes in provider behavior or components of behavior. An approach could be to frame this as a meta-epidemiological analysis, or a qualitative synthesis, comparing A&F trials with and without equity components in terms of outcomes such as provider behavior or even patient-level health outcomes. | Thank you for this comment. This would be valuable to include, however, this was not our research question. At the outset, we did not know if we would find any studies that were equity-oriented. As mentioned previously, we hope this work encourages others to do more equity-oriented analyses as part of their A&F initiatives, and have emphasized this in our discussion. Additionally, we anticipate that there would be much variability within equity-oriented studies regarding intervention components, equity concerns, and populations, that a comparison of outcomes would be biased and/or have inaccurate meaning. We have emphasized this challenge in our limitations as well. | 18, 19 |
| Introduction (Lines 79–81):  It would be helpful to specify how equity factors contribute to systematic health inequalities. For example, is it through differences in availability of services, accessibility, exposure to different risk factors, or other mechanisms? | Thank you for this comment. Specific examples have been added to the introduction. | 4 |
| Methods:  In Table 1, based on the stated objective, the appropriate comparator should be A&F trials without equity components. | Table 1 describes the selection criteria for studies included in the updated systematic review. As this was a secondary analysis, we did not have a study selection criterion. This analysis looked at all the studies in the updated systematic review. We have made this clearer in the methods (lines 136-141), | 7 |
| Discussion:  It would be helpful to clearly state whether it is assumed that A&F interventions with equity components demonstrate different efficacy in addressing equitable outcomes compared to standard A&F. Or does the efficacy of A&F itself depend on equity factors (i.e., an interaction effect)? | This secondary analysis was an exploratory first step to see if A&F interventions were considering equity and how. As this was not part of our research question, we have included an emphasis to focus on this in the future, in our discussion. | 19 |
